# A framework to simplify paediatric syncope diagnosis

**DOI:** 10.1007/s00431-023-05114-w

**Published:** 2023-07-20

**Authors:** Julian M. Stewart, J. Gert van Dijk, Seshadri Balaji, Richard Sutton

**Affiliations:** 1 New York Medical College, Valhalla, NY USA; 2 Department of Neurology, Leiden University Medical Centre, PO Box 9600, 2300RC Leiden, The Netherlands; 3 Oregon Health & Science University, Portland, OR USA; 4 Department of Cardiology, National Heart & Lung Institute, Hammersmith Hospital Campus, Imperial College, London, UK

**Keywords:** Diagnosis, Syncope, Childcare, Unconsciousness, Seizures, Risk

## Abstract

This paper aims to improve the diagnosis of syncope and transient loss of consciousness (TLOC) in children. Diagnostic problems stem, first, from some causes spanning various disciplines, e.g. cardiology, neurology and psychiatry, while the most common cause, vasovagal syncope, is not embraced by any specialty. Second, clinical variability is huge with overlapping signs and symptoms. Third, the approach to TLOC/syncope of the European Society of Cardiology (ESC) is underused in childcare. We explain the ESC guidelines using an additional paediatric literature review. Classification of TLOC and syncope is hierarchic and based on history taking. Loss of consciousness (LOC) is defined using three features: abnormal motor control including falling, reduced responsiveness and amnesia. Adding a < 5 min duration and spontaneous recovery defines TLOC. TLOC simplifies diagnosis by excluding long LOC (e.g. some trauma, intoxications and hypoglycaemia) and focussing on syncope, tonic–clonic seizures and functional TLOC. Syncope, i.e. TLOC due to cerebral hypoperfusion, is divided into reflex syncope (mostly vasovagal), orthostatic hypotension (mostly initial orthostatic hypotension in adolescents) and cardiac syncope (arrhythmias and structural cardiac disorders). The initial investigation comprises history taking, physical examination and ECG; the value of orthostatic blood pressure measurement is unproven in children but probably low. When this fails to yield a diagnosis, cardiac risk factors are assessed; important clues are supine syncope, syncope during exercise, early death in relatives and ECG abnormalities.

*Conclusions*: In adults, the application of the ESC guidelines reduced the number of absent diagnoses and costs; we hope this also holds for children.

**Table:** 

**What is Known:** *• Syncope and its mimics are very common in childhood, as they are at other ages.* *• Syncope and its mimics provide considerable diagnostic challenges.*	
**What is New:** *• Application of the hierarchic framework of transient loss of consciousness (TLOC) simplifies diagnosis.* *• The framework stresses history-taking to diagnose common conditions while keeping an eye on cardiac danger signs.*

**What is Known:**

*• Syncope and its mimics are very common in childhood, as they are at other ages.*

*• Syncope and its mimics provide considerable diagnostic challenges.*

**What is New:**

*• Application of the hierarchic framework of transient loss of consciousness (TLOC) simplifies diagnosis.*

*• The framework stresses history-taking to diagnose common conditions while keeping an eye on cardiac danger signs.*

## Introduction

Syncope is common at all ages. Approximately 40% of all people experience at least one syncope in their life [1]. Syncope constitutes up to 3% of emergency department visits [1]. Syncope care is inefficient with inappropriate costly tests and admissions [1, 2]. A diagnosis is often not made, due to a limited overview of possible causes and inappropriate evaluation. Diagnostic failure is explicable since syncope and its mimics may be due to cardiological, neurological, medical or psychiatric disorders. Diagnosis is made even more difficult, firstly by inconsistent definitions that incorrectly include concussion, epilepsy, metabolic disorders, stroke, transient ischaemic attacks and other conditions such as syncope in adults and children [3–5]. Secondly, some classifications ignore helpful features such as the duration of unconsciousness [6].

Realization of such problems led the European Society of Cardiology (ESC) to produce multidisciplinary syncope guidelines [1] comparable to North American ones [7]. The resulting diagnostic strategy reduced the proportion of undiagnosed cases, increased the number of diagnoses of vasovagal syncope (VVS) and decreased inappropriate tests and costs [1].

Unfortunately, these guidelines appear to have had a limited impact on paediatric syncope care [1, 6]. A recent systematic review revealed so many definitions and classification problems that the authors called for more diagnostic consistency [4].

This paper aims to facilitate paediatric syncope diagnosis by focussing on the ESC hierarchical approach to childcare [1, 8]. As therapy follows diagnosis, it is not discussed, except for a brief mention of nonpharmacological measures.

## Definitions

ESC definitions were formulated for pragmatic diagnostic utility (Table 1) [1], starting with clues to recognize the apparent loss of consciousness (LOC) through history-taking (Table 2), requiring three obligatory features:

1. Loss of motor control: this always comprises a tendency to fall but also has variable features: tone is flaccid or stiff, eyes are open or closed, limbs and neck may be flexed or extended, myoclonic jerks are present or absent
2. Loss of response to touch/speech
3. Amnesia for the period of LOC

**Table 1:** Hierarchical questions to establish a syncope diagnosis

**1. Is there an apparent loss of consciousness (LOC)?**
a. LOC requires non-responsiveness, abnormal motor control with falling, and amnesia
All three aspects must be present
b. Counter-examples not meeting all three criteria
a. Sleep-responsive
b. Narcolepsy/cataplexy—no amnesia
c. Absence/focal seizures with altered awareness—no loss of postural tone
**2. Is LOC transient and resolving spontaneously?**
a. Transient loss of consciousness (TLOC): LOC of short duration with spontaneous complete recovery
b. Counter-examples of longer-lasting or non-transient LOC
a. Coma
b. Intoxications
c. Metabolic (e.g. hypoglycaemia)
c. Counter-examples with recovery due to resuscitation
a. Promptly resuscitated sudden cardiac death
b. Promptly resuscitated from a potentially lethal drug overdose
**3. Which type of TLOC?**
a. Traumatic brain injury (concussion)
b. Syncope = TLOC + global cerebral hypoperfusion
c. Tonic–clonic seizures
d. Psychogenic/functional TLOC
e. Other causes
**4. If it is syncope, which type of syncope is it?**
a. Reflex syncope
b. Syncope due to OH
c. Cardiac syncope

**Table 2:** Essential clinical determinants of apparent loss of consciousness (LOC)

**Determinants of apparent LOC**					
		**Amnesia for period of LOC**	**Nonresponsive**	**Tendency to fall as an expression of abnormal motor control**	**Remarks**
**Apparent LOC**	Apparent LOC (see Table 3)	+	+	+	
**Not apparent LOC**	Absence seizures in children and focal seizures with altered awareness in adults*	+	+	−	
	Cataplexy	−	+	+	Almost always also excessive sleepiness
	Carotid TIA/stroke	−	−	Possible	Always neurological deficit
	Vertebrobasilar TIA/stroke	Very rare	Very rare	Possible	Always neurological deficit (temporarily in TIA)
	Falls	−	−	+	Fall may lead to true LOC through a concussion Patients may not report a gap in memory even with LOC. Without an eyewitness account, LOC can be mistaken for a fall without LOC

The presence of loss of consciousness usually must be determined after the fact through history taking, explaining ‘apparent’ (this is usually omitted, leaving ‘LOC’). The presence of ‘apparent LOC’ requires that all three features be present. If at least one feature is absent with certainty, apparent LOC is excluded. If one or more features are uncertain, apparent LOC must be considered

*LOC* loss of consciousness, *TIA* transient ischaemic attack

The presence of all three features guarantees that patients *appear* unconscious but do not prove gross cortical dysfunction as in syncope or generalized seizure, thus retaining functional causes.

**Fig. 1:**
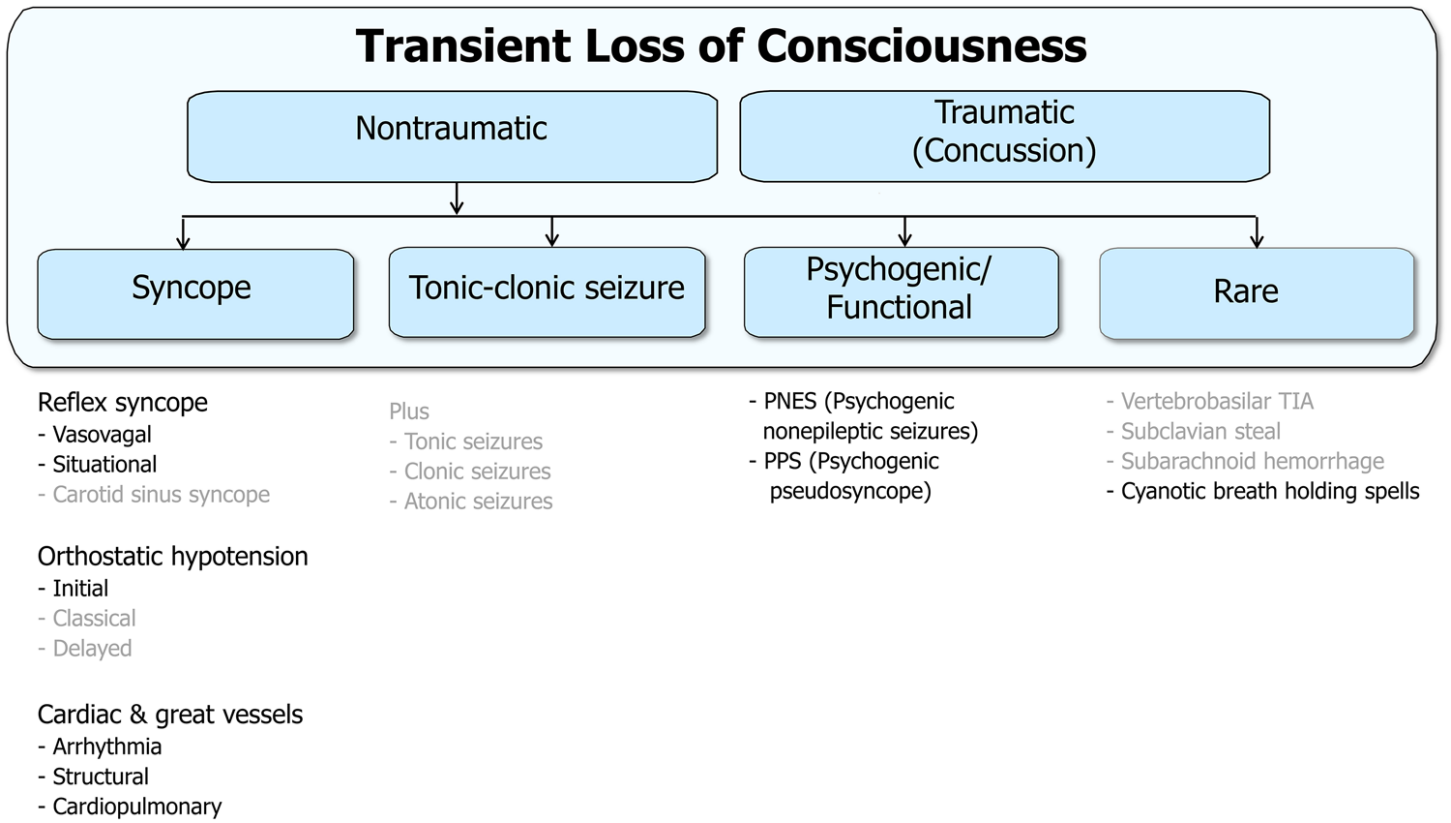
Classification of TLOC. Transient loss of consciousness (TLOC) is first divided into traumatic and nontraumatic forms. The nontraumatic forms cause most diagnostic confusion. There are four categories, each of which can be divided. Disorders that are rare in children are stated in grey as a reminder that the classification holds for all ages

The next step defines the major category ‘Transient Loss of Consciousness’ (TLOC) as LOC of brief duration with spontaneous recovery [1, 8]. Including eyewitness overestimation, a pragmatic maximum duration for TLOC is 5 min. Separating TLOC from other causes of LOC dramatically reduces the number of causes, mainly by excluding long-lasting LOC (Table 3, Fig. 1). To stress that the TLOC classification applies to all ages, all forms of TLOC are shown, but forms of little relevance to childcare are shown in grey and are not discussed in detail.

**Table 3:** Modifiers of apparent LOC

		**Duration of LOC**	
		Short (up to 5 min)	Long
**Mode of recovery from LOC**	Spontaneous recovery	**Transient loss of consciousness (TLOC)** Some concussions Nontraumatic: Syncope Tonic–clonic seizures Psychogenic TLOC Rare conditions	Some concussions Hypoglycaemia Intoxications Metabolic conditions
	Recovery through medical intervention	Aborted sudden cardiac death	Hypoglycaemia Intoxications Metabolic conditions

This table starts with ‘Apparent LOC’ as defined in Table 2. The two modifiers that create the category ‘Transient Loss of Consciousness’ (TLOC) are a short duration and a spontaneous recovery, both established through history taking. The presence of medical interventions can usually be established with certainty, but if the duration cannot be established reliably, both short and longer LOC must be considered

Syncope is defined pathophysiologically as TLOC due to global cerebral hypoperfusion [1] and not as a set of signs and symptoms, simply because no such set includes all syncope while excluding all epilepsy and psychogenic TLOC [9]. For instance, with syncope, facial colour may be pale, red, blue or normal; tone may be stiff or flaccid; movements may be present or absent. The resulting overlap of clues explains why syncope, epileptic seizures and functional TLOC are often mistaken for one another. Distinguishing them hardly ever rests on one clue, but instead on patterns of triggers, signs and symptoms [1, 8].

Diagnosing syncope requires a hierarchical procedure assessing the sequence of LOC, TLOC, syncope and type of syncope (Table 1) to achieve the most specific diagnosis the data permit.

Syncope partially overlaps with ‘orthostatic intolerance’, i.e. complaints while standing due to circulatory problems, as standing plays a role in vasovagal syncope and orthostatic hypotension. In postural orthostatic tachycardia syndrome (POTS), syncope appears no more common than in the general population and is commonly VVS [10].

## Diagnosis

The diagnosis of TLOC and syncope consists of the initial evaluation, risk assessment and specific investigations, if needed [1].

### Initial evaluation

History taking, physical examination, orthostatic blood pressure (BP) measurement, and ECG comprise the initial evaluation. History taking is crucial, first because circumstances, signs and symptoms provide crucial clues [1, 8], and second because tests performed in between spells are often normal. The most relevant clues are presented below; more are presented in the ESC practical guidelines [8]. ECG is rarely abnormal but warranted because of the rare chance of finding high-risk cardiac disease. The utility of conventional BP measurements before and after standing up in paediatric syncope is unknown but probably low. If no probable diagnosis can be made, risk assessment is required.

### Risk assessment

Risk assessment (Table 4) aims at detecting cardiac disease [1]. Questions assessing risks can easily be combined with the initial evaluation (see below) [11]. High-risk cases should be admitted to the hospital, although in some cases, patients may be discharged with close follow-up, provided the patient and relatives understand the need to avoid triggering syncope, for instance by avoiding competitive sports. Low-risk cases do not need immediate admission or testing. Cases with intermediate risk can be admitted for observation or referred to a syncope unit [1, 12].

**Table 4:** Clinical characteristics useful in risk assessment

**Cardiac**	**Non-cardiac**
**Historical features**	
Presence of known arrhythmic, structural, or ischemic heart disease	Absence of known arrhythmic, structural, or ischaemic heart disease
Family history of inherited cardiac disease or early sudden cardiac death	Absence of family history of lethal cardiac conditions. Presence of family history of VVS
Sudden onset with a brief or absent prodrome No autonomic activation before syncope	More gradual onset with a prodrome of autonomic activation, e.g. sweating, nausea, pallor
Syncope during or just after exertion Can occur with any posture, including supine	Prolonged standing (VVS and cOH), pain, fright (VVS) Sometimes after exertion (VVS, cOH)
Infrequent prior episodes	History of similar previous episodes
Less dependent on ambient conditions	Potentiated by heat, crowding, dehydration
Commonly not provoked; when provoked, anger, water in the face, loud noises, fever suggest inherited arrhythmia	Commonly provoked: pain, fear, prolonged standing, post-prandial, cough or laugh, micturition, deglutition, defaecation, stretch, hair-grooming
Aborted sudden death is present when LOC was ended by resuscitation	Spontaneous resolution is a defining characteristic of syncope
Use of QT-prolonging drugs (a useful website is: https://www.crediblemeds.org/*)* Drugs reducing potassium, calcium or magnesium, pro-motility drugs such as cisapride, Class IA and III antiarrhythmic drugs, SNRI antidepressants, macrolide antibiotics, fluconazole and related anti-fungals, 5-HT3 antagonists, antipsychotic drugs	-
**Physical examination**	
Cardiac examination may be abnormal	Normal cardiac examination. cOH may be present
**ECG**	
Bifascicular Block, Mobitz II AVB	Mobitz I AVB as a sign of enhanced parasympathetic activity (e.g. athlete)
Inappropriate sinus bradycardia	VVS incidence increased in athletes who typically have a slower-than-average sinus rhythm
Non-sustained ventricular tachycardia	≤ 3-beat monomorphic VT is normal
Long or short QT	-
Pre-excitation	-
ST segment elevation with Brugada type 1 morphology in V1-V3	Narrow QRS (< 0.12 s) no interventricular conduction delay
Ventricular hypertrophy	J point elevation with increased LV forces is a normal variant

## Forms of TLOC

### Concussion

Head trauma-causing TLOC must be distinguished from TLOC-causing trauma by asking which came first. TLOC-causing concussion may complicate diagnosis if traumatic amnesia obscures memories of the event.

### Syncope

The three major syncope groups are reflex, orthostatic hypotension and cardiac (Fig. 1). Triggers, posture and circumstances always play major roles, so a good first question is ‘what were you doing at the time?’. This should ascertain body position (lying, sitting, standing), the time spent in that position and the presence of any triggers.

There are two types of symptoms of impending syncope. The first is related to cerebral hypoperfusion, with visual changes (darkening, spots, loss of colour), hearing changes (distant sounds, tinnitus), light-headedness and LOC. These help establish syncope but not its cause. The second group suggests a cause, e.g. autonomic activation suggests reflex syncope (see below) and palpitations or chest pain suggest cardiac syncope.

As said, the tone may be stiff or flaccid, limbs may be flexed or extended and facial colour may be blue or pale, but the eyes are nearly always wide open during LOC [1, 8]. While the absence of jerking movements excludes tonic–clonic seizure, their mere presence does not differentiate syncope from seizure. Instead, their nature and number are useful: there are typically < 10 arrhythmic jerks in syncope and > 20 rhythmic jerks in tonic–clonic seizures [13].

Recovery from LOC in syncope typically only takes ~ 10 s. However, patients may feel fatigued/sleepy for hours afterward. Children may enter a deep sleep after syncope, usually after first regaining consciousness, unlike sleep in epilepsy which is often a continuation of LOC.

#### Reflex syncope

**Fig. 2:**
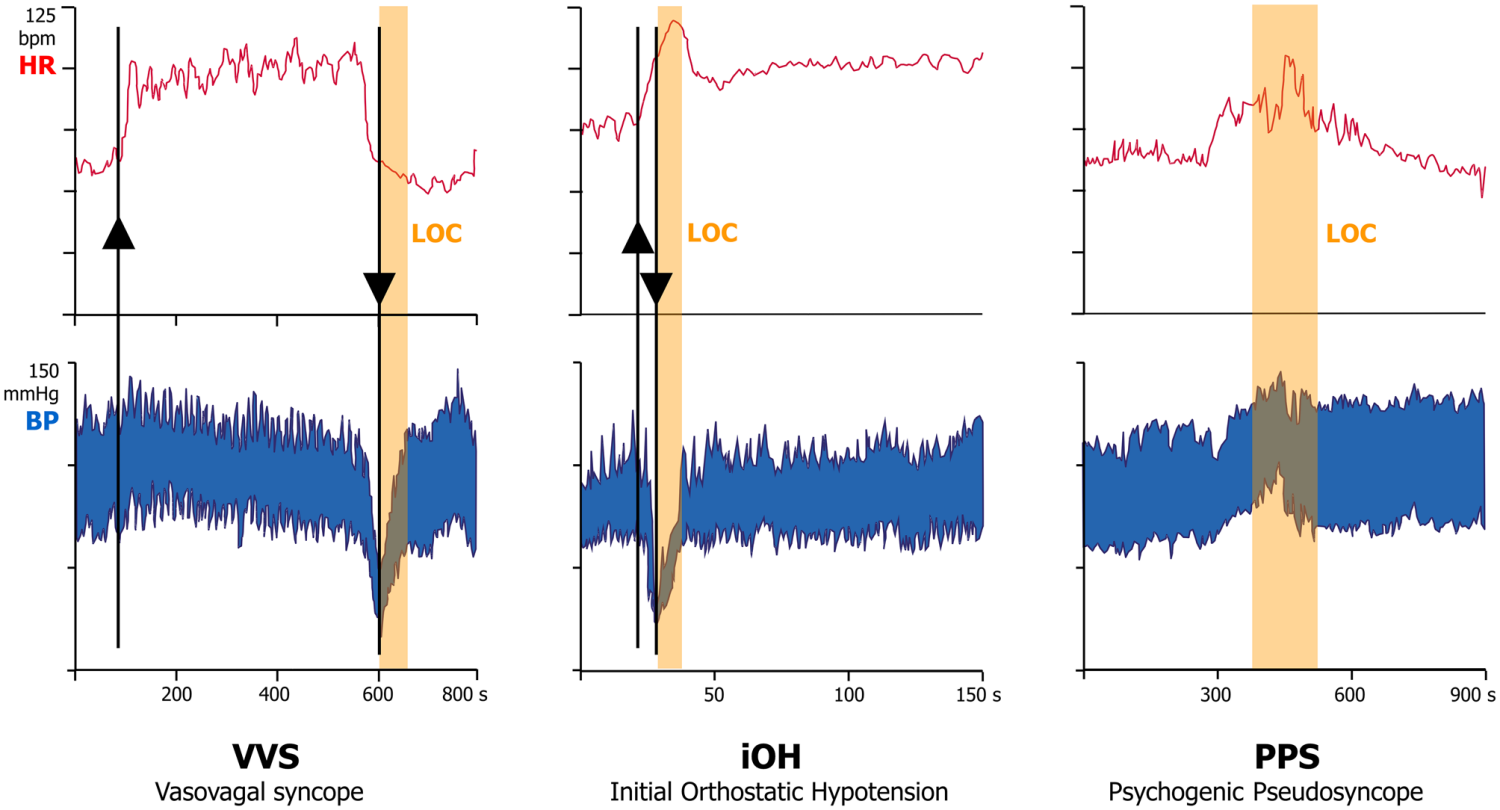
Haemodynamic patterns of common forms of TLOC. The three panels show heart rate (HR) and blood pressure (BP) for vasovagal syncope (left, VVS), initial orthostatic hypotension (middle, iOH) and psychogenic pseudosyncope (right, PPS). Orange bars represent the duration of apparent loss of consciousness (LOC)]. The upwards arrows indicate head-up tilt for VVS and active stand for iOH. Downwards arrows indicate tilt down for VVS and falling for iOH. The vertical scales for HR and BP are the same for the VVS, iOH and PPS panels, but the time scale differs. LOC in VVS is accompanied by low HR and low BP; in iOH by low BP and normal or high HR; in PPS, apparent LOC is accompanied by normal to high HR and BP. The BP nadir of VVS usually occurs many minutes after assuming the upright position, whereas this is a matter of seconds in iOH

In reflex syncope, a trigger evokes ‘autonomic activation’ before LOC, comprising sweating, pallor, nausea, and rarely vomiting or diarrhoea. The circulatory response consists of two parts, both reducing blood pressure (Fig. 2):

1. Vasodepression, i.e. vasodilation in muscle and splanchnic vascular beds causing venous pooling.
2. Cardioinhibition, i.e. a vagal heart rate fall due to sinus slowing that may include asystole of usually < 20 s. Cardioinhibition decreases with age [14] but is very common in childhood. Asystole is probably ubiquitous in VVS in toddlers. Asystole during reflex syncope is not a cardiac emergency, and there is no evidence of a poor prognosis [15].

Reflex syncope comprises three subgroups: vasovagal syncope, carotid sinus syncope and situational syncope (Fig. 1).

##### Vasovagal syncope (VVS)

Triggers are prolonged standing (usually > 5 min) or emotional through pain or fear; syncope is aided by crowded environments, changes in temperature, heat (external or internal, i.e. fever), dehydration due to excessive loss or decreased intake (sweating, vomiting/diarrhoea, fluid avoidance) and stress [1]. Hyperventilation facilitates VVS but does not alone cause VVS. The combination of typical VVS triggers plus autonomic activation without high-risk signs makes VVS so likely that no tests are needed beyond an ECG as a safety measure [1]. Chest discomfort and palpitations are common in adolescents, possibly related to hyperventilation, and are unreliable discriminators for cardiogenic syncope. Postsyncopal fatigue/sleep and headache are common [16].

VVS is by far the most common form of paediatric syncope with a peak age of onset of ~ 15 years [17]. The chances of VVS decrease thereafter but increase over the age of 60. VVS occurs twice as often in female than male adolescents [17]. Syncope recurs in at least 1/3 of VVS patients. Attacks vary widely in presentation and frequency.

##### Carotid sinus syncope

Carotid sinus syncope is so rare in the young that it need not be considered in paediatrics.

##### Situational syncope

‘Situational syncope’ refers to reflex syncope with a specific trigger such as swallowing, defaecation, micturition and coughing. There is no fundamental pathophysiological difference from VVS. Two forms preferentially occur in adolescence: hair grooming syncope [18] and stretch syncope, induced by stretching arms and neck upwards and backwards [19].

#### Syncope due to orthostatic hypotension (OH)

In all three OH forms discussed below, the circulation fails to maintain BP while standing. VVS is also often triggered by standing, but autonomic activation is present in VVS and absent in OH [20]. There are almost always multiple triggers in a patient with VVS, including pain, whereas only standing evokes syncope in OH. BP is low in OH and VVS, but heart rate is low in reflex syncope and high or normal in OH [20].

##### Initial orthostatic hypotension (iOH)

iOH is very common in adolescents. In iOH, BP decreases immediately after standing and spontaneously recovers while patients remain standing (Fig. 2). Symptoms range from mild light-headedness to syncope with falls. The typical history is standing up from a chair, walking three or four steps and falling. Syncope develops so quickly that the last memory may be standing up and the next lying on the floor. Conventional BP devices are too slow to capture the rapid changes of iOH, requiring specific ‘beat-to-beat’ monitors. Abnormality thresholds [1, 7, 20] include a decrease of systolic BP > 40 mmHg and/or diastolic BP > 20 mmHg within 15 s of standing. iOH occurs in its fully developed form in 15–20% of cases and in attenuated form in ~ 50% of all children [21]. Symptoms can be prevented by ‘counter-manoeuvres’ [21, 22].

##### Classical and delayed orthostatic hypotension (cOH and dOH)

cOH and dOH are defined as a sustained fall in blood pressure while standing [1, 8]. The rarity of chronic cOH in children [23] suggests VVS and iOH as more likely causes of syncope during standing. When cOH is present, its causes parallel those in adults, including medication, type I diabetes and hypovolemia [23].

#### Cardiac syncope

##### Signs and symptoms

Cardiac syncope carries intermediate to high risk. Supine syncope is a cardiac danger sign because it suggests circulatory standstill: in most reflex syncope and OH, the circulation does not stop altogether, so lying or sitting alleviate complaints. When these do not help, a circulatory standstill implying cardiac syncope is more likely. VVS evoked by needles may well occur in patients already lying down and is then usually due to asystole causing a circulatory standstill, but not a dangerous one. Also note that epileptic seizures and functional TLOC may occur while lying. Syncope during exercise is also a danger sign, although this can also occur in VVS. Syncope just after exercise also carries risks: while occurring often in VVS (and cOH), it also occurs in arhythmic syncope [24]. Exercise stress testing is essential. Always ask for unexpected deaths in family members of < 40 years, as these suggest inherited cardiomyopathy or arrhythmia [1].

An ECG is essential, as many cases of cardiac syncope occur in existing although undetected structural heart disease. Most ECG abnormalities are relevant, although evidence of ischemia is rare in paediatrics (anomalous coronary artery). Echocardiography is necessary if the history suggests cardiac syncope, even with normal ECG.

##### Arrhythmia

Syncope from primary arrhythmia is typically due to abnormally high heart rate (‘tachyarrhythmia’), rarely due to low HR (‘bradyarrhythmia’). Diagnosis can be made by extended external or internal ECG loop recording (ILR) [25]. When myocardial function is normal, HR must be extremely high for syncope to occur, although standing increases the chance, by decreasing venous return. Most paediatric supraventricular tachycardias (SVT) and many ventricular tachycardias (VT) are tolerated with mild symptoms and palpitations. In contrast, very rapid VT, as seen with many channelopathies, cause syncope if lasting more than 3–5 s. There are typically no prodromes, so LOC occurs without warning. If a tachyarrhythmia with syncope reverts spontaneously to sinus rhythm, consciousness is rapidly regained. When episodes last < 30 s, the patient appears normal immediately and asks, ‘What happened?’ without recall of onset; this suggests an arrhythmic cause. If LOC due to arrhythmia is ended by resuscitation, this should be termed ‘aborted sudden cardiac death’, not syncope, as underlying causes differ.

Arrhythmic syncope in children and adolescents is often due to genetic causes. The resting ECG may contain clues, such as prolonged/abnormal QT interval. Inherited arrhythmic syncope may have specific triggers, e.g. startle such as by loud noise or cold water on the face (long QT syndrome), sudden onset syncope during fever (Brugada syndrome) and syncope during exercise/excitement (catecholaminergic polymorphic VT or arrhythmogenic right ventricular cardiomyopathy) [8].

##### Structural cardiovascular disease

Structural disease can cause syncope if cardiac output becomes insufficient to meet metabolic demands. Echocardiography is required. In children, causes include aortic stenosis, coarctation of the aorta, anomalous coronary arteries, tetralogy of Fallot and transposition of great arteries. Many congenital heart diseases especially those with a ventricular surgical scar can trigger arrhythmia, and residual heart disease can trigger cardiac syncope. Conditions presenting more commonly in adolescence are primary pulmonary hypertension and hypertrophic or dilated cardiomyopathy.

### Epileptic seizures

Syncope is the most common cause of misdiagnosis of epileptic seizures [26], with futile anti-epileptic medication as a serious consequence.

### Not all seizures cause TLOC

LOC in syncope and tonic–clonic seizures is due to a global cortical dysfunction [27], while the dysfunction is only partial in other seizure types. A fundamental clue is whether patients remain upright: in the absence of seizures of childhood and some focal seizures, responsiveness and memory are affected, but patients remain upright, which excludes TLOC. Only epilepsy types that cause falls result in TLOC, tonic–clonic seizures being the most common.

### Clinical features of tonic–clonic seizures

Tonic–clonic seizures are rarely triggered but often have specific prodromes, the aura. Auras differ between patients but are stereotypical within patients: déjà vu and unpleasant smell or taste are common. During the aura, patients may show abnormal responsiveness but are upright, so this phase is not TLOC; TLOC then starts with the seizure becoming generalised, which may start with a cry and a stiff fall. Eyes are wide open as in syncope. There are 20–100 repetitive jerking movements [13]. A lateral tongue bite is specific (in syncope the tongue tip may be bitten) [1, 8]. The face may turn blue as in cardiac syncope. Once jerks are over, patients may enter deep sleep for many minutes. Once awake, patients do not comprehend the situation and may repeat questions, in contrast to syncope. Urinary incontinence, eye-opening, fatigue and sleep afterwards occur in syncope and seizures and thus are of limited diagnostic use [8].

### Seizure-causing syncope and vice versa

In ‘ictal asystole’ cortical seizure activity causes asystole. A focal seizure develops as typical for that patient but suddenly ends with a fall with complete unconsciousness, i.e. syncope [28, 30]. Ictal asystole can occur at any age [29] but happens only in a fraction of a patient's seizures [30].

Syncope in infants can rarely trigger a tonic–clonic seizure; these events have been termed ‘anoxic epileptic seizures’ [31].

### Psychogenic/functional TLOC

There are two types: in psychogenic pseudosyncope (PPS), patients lie still, superficially resembling syncope, while jerking movements resemble seizures in psychogenic nonepileptic seizures (PNES) [1, 8].

Strong clues are frequent episodes (many/week or daily), long (> 10 min) attacks and consistently closed eyes [32]. Physical injury does not exclude psychogenic TLOC. During spells, blood pressure and heart rate are likely to be high rather than low [32] (Fig. 2), and EEG and cerebral blood flow are normal [1, 32]. Documenting the nature of attacks increases diagnostic accuracy [33]. Video recording of a spell is often successful because of the high frequency and long duration of attacks, allowing scrutiny of muscle tone, movements and eye closure. Provoking a spell in the hospital allows documentation of physiological signals. For PNES, video-EEG is the gold standard, while for PPS, this is tilt testing with video-EEG, heart rate, blood pressure or records of cerebral perfusion [34]. Both types occur in adolescence and young adults and are more frequent in females than males. Attacks are often not triggered and can occur when supine, and there is no autonomic activation. In PPS, body position resembles sleep. Facial colour is normal, and patients lie more still than in VVS [32]. Patients with PPS often also have VVS and patients with PNES frequently also have epileptic seizures.

## Other TLOC forms

### ‘Reflex anoxic seizures’ and ‘breath-holding spells’

Both terms refer to TLOC in infants/toddlers. The word ‘seizure’ in reflex anoxic seizures may suggest epilepsy, but the spells are in fact cardioinhibitory VVS with asystole, evoked by unpleasant or surprising stimuli. To avoid confusion, ESC Guidelines recommend ‘VVS in infants’ [1, 8].

A typical episode comprises a trigger, a fall, no cry, a child lying still, eyes open and upwards, stiffening with rigid extension and a few jerks [35].

Pallid and cyanotic types of breath-holding spells describe facial colour during an attack [36]. Both start at ~ 10 months of age. Pallid breath-holding is synonymous with ‘reflex anoxic seizures’, i.e. VVS in infants [37], so ‘breath-holding’ is a misnomer for these events. In contrast, respiration plays a role in ‘cyanotic breath-holding spells’ [38]. Startle or sudden hurt causes a reflex to stop respiration in expiration involuntarily, with secondary circulatory impairment. Reports of pallid and cyanotic spells occurring in the same child [39] suggest the two types are not mutually exclusive.

### Other TLOC forms

True short-lived LOC can occur in subarachnoid haemorrhage and other rare disorders in adults, but these are rare in childcare.

### Notes on therapy of iOH and VVS

Nonpharmacological means to prevent syncope are the mainstay of therapy of iOH and VVS and should be taught to all patients; they are explained on page e74 of the ESC Guidelines Practical Instructions [40]. None has been proven in children. For VVS, long-term increased water and salt intake or bolus water ingestion for risk situations (e.g. standing a long time) aims to prevent low blood pressure. When syncope is imminent, lying down is the best option, followed by sitting or manoeuvres to increase blood pressure, such as forceful leg crossing. Specific iOH measures are not to walk away immediately after standing up but to wait and to sit down or perform leg-crossing if symptoms appear [21, 22].

Instructing the correct performance of such measures should be part of teaching physicians about syncope, further helped by the practical instructions [40], free webmaterial along ESC guidelines^1^ or courses to improve communication about syncope [41].

## Conclusions

A hierarchical framework to diagnose syncope is presented, resting on the determination of first LOC, then TLOC, syncope and the type of syncope. This framework, common to all specialties, encourages a broad diagnostic view, comprising all forms of syncope, epileptic seizures and psychogenic TLOC. Vasovagal syncope may be considered as much a part of paediatric medicine as it is part of cardiology, neurology and other specialties. The use of a common framework improves diagnosis and care at all ages.

## Abbreviations

BP: Blood pressure
cOH: Classical orthostatic hypotension
dOH: Delayed orthostatic hypotension
ECG: Electrocardiogram
EEG: Electroencephalogram
ESC: European society of cardiology
ILR: Implantable loop recorder
iOH: Initial orthostatic hypotension
LOC: Loss of consciousness
OH: Classical orthostatic hypotension
PNES: Psychogenic nonepileptic seizures
PPS: Psychogenic pseudosyncope
SVT: Supraventricular tachycardia
TLOC: Transient loss of consciousness
VT: Ventricular tachycardia
VVS: Vasovagal syncope

## Footnotes

1 https://www.youtube.com/watch?v=Usjk_DYHcg0, https://www.youtube.com/watch?v=oSiiXk4GZ3U
